# Meeting July 6

**Published:** 1886-09

**Authors:** 


CHICAGO MEDICAL SOCIETY.

*Regular Meeting, July 6th, 1886.*—THE PRESIDENT, E. J. DOERING, M. D., in the chair.

PROFESSOR CHARLES T. PARKES reported a case of urinary fistula of twelve years' standing, from gunshot wound of thigh. The track of the bullet had been such that the urine could escape through the wound on the thigh. Several unsuccessful attempts had been made to close the fistula. While examining the bladder with a sound, Professor Parkes discovered a stone. Lithotomy was performed, and now the patient urinates *per urethram*, and the fistula is closing. The calculus was the size of a pullet's egg, and the nucleus consisted of a piece of bone. Professor Parkes also exhibited a thirty pound multicular ovarian cyst with colloid contents, which had been removed without enlarging the original incision. He also exhibited specimens of hydatids of the abdomen, connected with liver, spleen and intestines. Exploratory operation had been made, but the hydatids were so numerous, large and adherent that they could not be removed. The patient died of exhaustion. One hydatid of the spleen measured twenty-three inches in circumference.

PROFESSOR W. T. BELFIELD said, the case which particularly interested him is the one in which the stone was found in the bladder, and he congratulated Professor Parkes on his discovery of that stone, because the case had been a reproach to surgery for twenty years. He had once seen the patient, who was kind enough to perform for his benefit, and threw a stream some little distance through the fistula in the thigh. Probably the stone was overlooked from the fact that it was situated pretty high up, attached to the upper wall of the bladder. Recently he had a case in which stones weighing two and one-half ounces had been carried by the man for eight years; he had been sounded a number of times, once under chloroform, but with negative result every time. In that case he found one of the stones attached to the fundus of the bladder, the other ensconced behind an enlarged prostate.

PROFESSOR R. G. BOGUE said: The cases presented are of a great deal of interest. Probably the most interesting feature of the case of stone in the bladder was the discovery of the offending body and its removal. The strange paths that bullets take in their course through the body, or a portion of it, were found to be so various by those who have had opportunities for observation during the war. The fact of this bullet passing into the side of the thigh, wounding the bladder and passing out through the perineum, was not so very strange; but the repair which took place in the track of the bullet, the lodgment of the large fragment of bone, such as we see here, within the bladder where it remained so long a time without giving a great deal of trouble, is somewhat singular. The long experience that the man had in seeking relief, or cure, without avail, and the probably accidental discovery of the real difficulty and its remedy by removal of the stone, renders the case one of a a good deal of interest. The case of hydatid cysts is also of much interest. Hydatid cysts are supposed to be comparatively rare, although in hospitals where a great many post-mortems are made they are frequently found. In many instances they give so little trouble during their growth that they are only found upon post-mortem examination as an accidental affair, the liver being the seat of the great proportion of instances of their development. In looking over the first volume of the catalogue of the museum of St. George's Hospital, of London, he found that there are eighteen specimens of hydatid cysts mentioned, one of the lungs in a lad who had had chronic cough for some two years, which subsided after he had expectorated an hydatid cyst. All the other hydatids developed from the liver; one was a case of multiple cyst where a large cyst had developed upon the superior surface of the liver, causing absorption of the diaphragm, entering the thoracic cavity and becoming adherent to the right lung; the abdomen was quite filled with tumors attached to the omentum, the spleen, the kidney, and the under surface of the liver, and some to the peritoneal surfaces. In this case there had been, no doubt, a ruptured cyst, as the young man had fallen while at play, and the accident was followed by severe symptoms of prostration or collapse, with recognized peritonitis, from which he finally recovered, but afterwards died of erysipelas. The tumors in the abdomen had been diagnosticated some two years previously, but the death was from a cause other than that connected with the growths themselves, and the revelation upon postmortem was that of extensive hydatid disease, the greater part being connected with the liver. All of the other instances of hydatids were of the abdomen, connected with some portion of the liver, either within the organ or upon its surface, so that in all of these eighteen cases the specimens were from the liver or within the abdomen, having the liver for their primary seat, with the exception of the one in the lung.

PROFESSOR J. S. JEWELL said he had been deeply interested in listening to the cases reported by Professor Parkes, but especially in the case of urinary fistula. He thought in such cases as stone in the bladder, as well as all others, there should be the most thorough and careful investigation. When one undertakes to explore the bladder for stone, cases like this ought to be held in mind. He suspected that in the case in question Professor Parkes was pretty nearly giving up the examination with the sound when he made the last sweep that brought the stone to light. He was impressed with the necessity for more thorough work than is usually given in the matter of diagnosis. He was becoming more careful every day, as a matter of habit and duty. In studying cases, apparently the most simple, one often finds, where we least suspect it, remarkable things. He throught there was toomuch hap-hazard diagnosis to produce the best work, and this case is an example of it; for, if Professor Parkes could find the stone, others should have done so.

PROFESSOR C. T. PARKES, in closing the discussion, said: Professor Jewell is correct in his supposition that it was by a mere accident that he found this stone. Perhaps he had neglected to say, in regard to the hydatids, that there is no tissue in the body that is free from this parasite; there are only certain places in the body where it is found with the absence of an adventitious sac, but some, especially those found in the brain, are usually cysts with but two layers, the terminal membrane and the external layer. There are many things that seem to refer their formation and development to the circulation of the broad cells through the blood, and there are many things that seem to show that this cannot be possible.

DR. HENRY T. BYFORD read a paper entitled

THE MECHANICAL TREATMENT OF RETROFLEXION OF THE UTERUS.

The means for correction of the retroversion are of four kinds:

1. Those which permanently fix the fundus in front of the pelvic axis;

2. Those which draw or fix the os back of the pelvic axis;

3. Those which place a barrier or obstacle to the forward displacement of the os and cervix;

4. A combination of two or more of these methods.

The fixation of the fundus forward has been done in four principal ways:

1. By the Alexander operation, in shortening the round ligaments. It was suggested by Alquié, recommended by Aran, experimented upon on the cadaver by W. A. Freund, and successfully performed and established as a therapeutic measure by W. Alexander.

2. The stitching of one or both round ligaments to the abdominal walls; as has been done with permanent success by Wm. H. Byford while performing laparotomy for another purpose.

3. The stitching of one or both of the broad ligaments to the abdominal walls; as successfully done by Koeberle and

Schroeder, and perhaps others, during laparotomy for other pathological conditions.

4. The stitcing of the uterus to the abdominal wall, as recommended by Mueller and Tait, and performed by Heywood Smith by an especial laparotomy.

The drawing or holding of the cervix back has been accomplished by uniting the cervix to the posterior vaginal wall by adhesive inflammation or by operation, as recommended by Loewenthal. But the most available method is by the use of pessaries of the Hodge class, such as the Albert Smith, Thomas, Emmett, Hewitt, Hauks, Noegervath, Schroeder, Gehrung, etc., which hang up the cervix posteriorly and drop the fundus forward, and thus supplement or supplant the posterior suspensory ligaments. The modifications of the Priestly instruments, Cutter's, Lazarewitsch's, Thomas's and Scott's, are also useful. H. Marion Sims's new retroversion, Hodge and stem pessary may be classed here, although original in action. Peaslee's, Mayer's, Dumont-Pallier's, the inflated rubber rings and bags, etc., are to be taken out oftener than to be put in.

The cervix may be kept back by lubricated cotton plugs, changed every day, or by pessaries such as the Courty, the Gehrung retroversion and the author's new instrument, which consists of a thick crescent held in front of the cervix by being attached to arms which work on the lever principle. It looks something like a Thomas retroversion pessary bent so as to come in front of the cervix. It has been made to fulfill the following six requirements :

1. To place the uterus in a normal or nearly normal position ;

2. Not to interfere with the natural supports ;

3. To afford an elastic or yielding support;

4. Not to interfere with the use of a speculum;

5. Not to interfere with the marital relations;

6. The patient is able to introduce and remove it.

The Hodge varieties are generally faulty as to requirements 1, 2 and 6.

The patient in most instances has but to introduce the neck and shoulders of the instrument, assume the knee chest position and push it into place. It allows the vagina to collapse, and does not press injuriously upon the uterine supports.

Especial contra-indications to this form of pessary are: tenderness or induration in the vesico-cervical region, decided retroflexion, an insufficient projection of the cervix into the vagina, and an unusually short anterior wall of the vagina.

Especial indications are retroversion with subinvolution after abortion or labor, or with bilateral laceration of the cervix in which the traction of the other forms acts hurtfully, a lax vagina, post cervical tenderness.

In preparing a subinvoluted uterus with bilateral laceration and eversion but without retroversion, it is exceedingly useful for lifting the uterus off the pelvic floor. Lateroversion may be corrected by raising one shoulder of the instrument.

Dr. Byford also described and illustrated by charts *a new operation for raising the perineum and pelvic floor* in such a manner as to form an obstacle to the forward displacement of the os and cervix, and at the same time strengthen the vaginal connective tissue attachments. The denudations run across the muscular fibres of the levator ani in such manner as to enable the operator to shorten those fibres and thus lift up the perineum, posterior vaginal wall and pelvic floor, to the greatest possible extent with the smallest loss of tissue. A transverse strip is denuded just posterior to the fourchette or carunculæ, and a narrow triangle with its base at the posterior commissure, or behind it, and apex in the posterior vaginal wall, made to cross it. This makes a sort of star whose angles are sewed up, in the form of a cross, the transverse strip coming together as a transverse line, the triangle in a line at right angles to it and passing up the posterior wall of the vagina. Sometimes the vulval wedge or base of the triangle is entirely left out, and sometimes represented by a nick in the fourchette ; and sometimes the apex of the triangle is represented by two lateral strips passing to either side of the rectum, the latter for the purpose of getting at deeper fibres of the levator ani.

The size of the different parts of the figures must be determined by the case, and are usually very small in those who may be expected to bear children afterwards. The denuded areas must sometimes be altered so as to remove cicatrices and repair particular injuries, the principle of the operation being to produce a natural elevation and condition of the parts, instead of constructing an artificial or unnatural body or cicatrix. In cases of unusual difficulty the uterus should be anteverted and the anterior vaginal or cervical wall be united to the posterior vaginal wall where they meet, and the operation for raising the perineum and pelvic floor then performed.

The Fitch and Studley, Schultze's figure eight and sleigh pessaries, the Hurd, Fowler, Fritsch and Woodward are cited as examples of tractions behind combined with pressure in front of the uterus. Many of them are faulty in that they produce a rigid fixation of the cervix—and such should be avoided. Other combinations are mentioned.

The author recommends the mechanical treatment of retroflexions chiefly as an aid in their treatment, and would especially keep back operative measures as adjunctives, palliatives, last steps or resorts, or as the least among evils.

DR. H. P. MERRIMAN said he had been very much interested in the paper, which he thinks is a valuable one. It seems to deal not merely with the subject of pessaries, but with the various means of support in the case of retroversion. He believes that a great many physicians, when they find a retroversion, without stopping to consider its cause, at once feel that it is necessary to employ a pessary, and in a great majority of instances the use is followed by failure to relieve. We all know that retroversion of the uterus has more than one cause; it is due in a great many cases to pressure from above, to weight within the uterus itself, as in the case of a fibroid tumor; the use of the pessary in these cases is of no value —it is only when there had been a weakening of the supports. In the case of weakened ligaments the pessary is of value as a temporary expedient. When the retroversion is due to a weakened vaginal support, which is true in the great majority of cases, for when we find the perineum ruptured, even partially, we are going to have, sooner or later, a retroversion. We find pessaries valuable in these cases, though, as a rule, we should not depend upon them permanently, because we need to restore the vaginal supports by some kind of operation, such an operation as restoring the perineum and curing a rectocele or cystocele, or by the general operative procedures Doctor Byford has mentioned. He believes that what we need in nearly every case is to examine the vagina and restore it to its proper shape and position. The uterus is retroverted because the vaginal support is gone, the wall of the vagina has become relaxed and is letting down the uterus, and we want to restore that wall of the vagina. If there has been a ruptured perineum, restore the perineum; if there has not been, the uterus, and by keeping it in place for six months or a year regain the support of the rested vagina. This will be done in a little different way from what Dr. Byford has suggested. We have got to fit something to the vagina that will extend the posterior wall and push up the cul-de-sac back ot the uterus. We cannot do that where there is tenderness, or where there is a tumor; but where there is not, and it is merely a simple retroversion, then it will be necessary to fit the pessary to the vagina, and have it fit in such a way as to elongate and support the vagina in a natural shape. It always distresses to hear men speak of fitting a pessary to the uterus. He does not believe it should be fitted to the uterus. It should be fitted to the vagina: the object is to restore the vagina to its natural position, and we must choose a pessary especially adapted for that purpose, and it should lie easily in the vagina.

PROFESSOR WILLIAM H. BYFORD said he came here with the determination of not speaking upon this subject to-night, because the scope of the paper is so great that if he were to undertake to comment upon half the points it would take too long. He believes the principles of the paper are correct for the treatment of this form of displacement of the uterus, especially the one of acting upon the cervix. His impression is that in retroversion of the uterus there is stretching of the utero-sacral ligaments until they are relaxed, and we find connected with it relaxation of the vagina, which he thinks is more frequently the consequence than the cause.

DR. FRANKLIN H. MARTIN asked Dr. Byford what advantages he claims for his pessary over the German sleigh pessary? If the fulcrum of this pessary is as indicated in the large diagram, situated at a low point on the posterior vaginal wall, how is he going to get any support for his fulcrum in a case of lacerated perineum? His illustration represents the fulcrum resting very low in the vagina, and it would have no support if the perineum is even partially lacerated.

PROFESSOR T. D. FITCH said: Dr. Merriman thinks supports for the uterus are abused, and he agrees with him in that opinion. They are abused because practitioners do not take the trouble to enlighten themselves with regard to the use of these mechanical supports, but when they get a case that requires a mechanical support they go ahead thoughtlessly to adjust a pessary of the latest device to support a displaced uterus. If from different causes the uterus has become displaced, do not the uterine ligaments become weakened as the result of that displacement? You never have a case of displacement that the uterine supports do not become weakened and relaxed, and can you tone up a muscle or a ligament that is placed upon the stretch to its utmost capacity, by any means, while in this tense condition? It is impossible. We must assist those ligaments to regain their tone by these mechanical supports, relax the ligaments, give them rest, and then by local and general treatment give them tonicity. Having done this, you can remove your artificial supports. He also fully endorses what Dr. Merriman says with regard to fitting the pessary to the uterus. The pessary should conform to the normal form of the vagina, and that is why we have to have this flexible material so that we can bend them by heat and make them fit the different shaped vaginæ. He does not approve, as a rule, of the principle of leverage. And that is why so many physicians fail in the use of Hodge's pessary; the leverage is too great. The pressure is so great in using this leverage that abrasion occurs, and laceration and cutting through the tissues. Pessaries should never be fitted in such a way as to produce abrasion, laceration or cutting through the tissues. They should not press hard; they should distend the vagina to its normal length, especially, not its normal breadth, and this can be done without much pressure where the uterus is replaced so that the fundus falls forward so as to be in front of the transverse axis of the uterus at the junction of the cervix with the body. If it is thoroughly replaced, then there is not much pressure when the pessary is introduced. It requires little force to hold the cervix back, and he believes in the majority of cases that here is where the general practitioner fails, viz.: in getting the fundus thoroughly forward, and the uterus replaced. Many times it is half raised up and the pessary presses against the body of the uterus so hard that it will imbed its whole thickness in the body of the uterus, producing inflammation.

He has frequently held the sound in the uterus and held the uterus up thoroughly anteverted, or thoroughly at right angles with the vagina, and introduced the pessary over the sound so as to secure thorough replacement of the uterus. He believes in the use of the pessary, not only as a support to the uterus, but as a splint to the vagina; for if the vagina is kept in its normal position the uterus will necessarily be kept in its natural position. The ideal pessary, in his opinion, is the pessary of Hodge. Emmett's pessary will fit more vaginas than Hodge's or Smith's, the latter differing from Hodge's in that its vuval extremity is narrow instead of broad; Hodge's is broad, while Smith's and Emmett's are both narrow at the lower extremity and are supported by the walls of the vagina. Emmett's is much better than Smith's, is much larger, and therefore much less liable to press too hard upon tissues. The pessary of Dr. Byford, which he has introduced to-night, is the form which Professor Fitch has improvised extemporaneously for himself, and used in several cases. He had six cases where the tissues in the posterior vaginal junction were so sensitive that it was impossible to use a Hodge, Smith or Emmitt, and he took the ordinary pessary of Hodge or Smith, and bent it in the form of a Byford pessary, and found he could use it where he could not use the others. There is an objection to placing this pressure upon the anterior surface of the cervix with a firm, unyielding instrument, and he does not believe that Dr. Byford's pessary will entirely remove that difficulty. He has stated in his paper that there is, in a great many cases, an absence of the anterior lip of the cervix uteri; there is not sufficient of it to be received on this instrument and to be held; it slips off and down in front of the instrument. This is not the only objection. He has found that while the pressure is brought upon the anterior surface of the cervix by the edges on his instrument, it so interferes with the circulation that the anterior lip will become swollen and œdematous. In his instrument there is no ring for the cervix to become imprisoned upon; this is certainly a thing much to be desired where there is an ulceration or laceration existing. If the pressure could be divided between the posterior vaginal junction and the anterior surface of the cervix, the œdema would be much less than where the whole uterus was held up by the pessary. These pessaries, Byford's and his, are certainly very strongly indicated in cases where there is great tenderness in the cul-de-sac. A prolapsed ovary with a retroverted uterus may fall down into the cul-de-sac of Douglass and no pressure can be borne there at all, and in such cases the only pessary that can be used with success is one that brings the pressure to bear upon the anterior surface of the cervix uteri.

PROFESSOR SARAH H. STEVENSON said she would like to ask how to treat cases in which the fundus lies high and in which the pressure upon the surface has no effect whatever. Where the fundus is low there is no difficulty. It is very easy to cure that sort of retroversion, but where the fundus is high she does not know how to treat the case.

DR. HENRY T. BYFORD said, in closing this discussion, he thinks it is wrong to say that pessaries are fitted to the vagina; they may be fitted either to the vagina, uterus, or pelvic floor, or all three. In regard to the bearing of this instrument, it forms almost a semi-circle in which the cervix fits loosely and does not get directly pressed upon. It also makes a good support for a uterus that is not retroverted, but which rests on a pelvic floor. He has a case of bilateral laceration with eversion to the third degree, in which, after this pessary was applied to the extensive ulceration which was due to friction upon the pelvic floor, got well in three weeks. He has a case of fibroid tumor in which the uterus lay directly across the pelvis, the right horn on a level with the cervix, but which is held about straight by this pessary modified by having one shoulder lifted, thus giving the patient back her former comfort. In regard to Dr. Martin's question—in the case of laceration just mentioned the levator vaginæ portion of the levator ani seem ruptured or relaxed, and leaves a large vaginal outlet, and yet a good sized instrument is retained. A large instrument is of course required for a large uterus or a relaxed vagina. You can change the position of the fulcrum by changing the curve of the arms. The sleigh pessary if reversed looks very much like this one with the handle cut off, but it would require a change in the curve of the arms and in the neck before it could be similarly used. This pessary comes the nearest being a perfect representation of one of Professor Fitch's instruments, which he devised before he became sick, but has not exhibited until to-night, and which is a modified Courty's. About the operation, he would like to say that his object in performing it is merely to raise the natural tissues, not to build an artificial support or barrier of fanciful shape; not to remove any more tissue than is absolutely necessary, but to draw the tissues together as much as is possible or desirable. What he has tried to do has been to find the directions of the muscular fibres and shorten them a little, and if they have been torn to reunite them as they were originally. At the same time he has always in mind the gathering up of the loosened connective tissue about the denudation, and sometimes cuts a little deep at certain points in order to cut into it and make a closer union of tissues.

